# Algorithm for a particle-based growth model for plant tissues

**DOI:** 10.1098/rsos.181127

**Published:** 2018-11-28

**Authors:** Joost H. J. van Opheusden, Jaap Molenaar

**Affiliations:** Biometris, Department of Mathematical and Statistical Methods, Wageningen University, Droevendaalsesteeg 1, 6708 PB Wageningen, The Netherlands

**Keywords:** morphology, simulation, structure

## Abstract

We have developed an algorithm for a particle-based model for the growth of plant tissues in three dimensions in which each cell is represented by a single particle, and connecting cell walls are represented as permanent bonds between particles. A sample of plant tissue is represented by a fixed network of bonded particles. If, and only if a cell divides, this network is updated locally. The update algorithm is implemented in a model where cell growth and division gives rise to forces between the cells, which are relaxed in steepest descent minimization. The same forces generate a pressure inside the cells, which moderates growth. The local nature of the algorithm makes it efficient computationally, so the model can deal with a large number of cells. We used the model to study the growth of plant tissues for a variety of model parameters, to show the viability of the algorithm.

## 1. Introduction

The basic building blocks of all higher organisms are biological cells. The growth, morphology and functionality of the organisms are based upon the growth, division and interaction between the cells. An important distinction between plant and animal cells is that plant cells are connected to each other by the middle lamella, a pectin layer formed at cell division. Because it irreversibly glues the cells together, plant tissues consist of a permanent network of cells, the topological structure of which only changes upon cell division. This has serious implications for models describing plant growth from the cellular level.

The model we present for the developing cell network in plant tissue is based upon the molecular or Brownian dynamics simulation techniques from physics and chemistry. These techniques describe particles interacting via potentials. The equations of motion can be implemented very efficiently, allowing for large-scale simulations on relatively moderate computing equipment. To use this efficiency, in our model, we identify plant cells with particles in a similar fashion, to describe the growth of plant organs or even whole small plants *in silico*. Cell division is a phenomenon that is very specific to biological systems, and absent in molecular systems. The core of the modelling hence is finding a viable way of including the restructuring of the bonded network at cell division into the model.

As the mechanical properties of most plant tissues are largely determined by those of the cell walls, it is important to include the cell wall in the model. In our approach, the dynamics of the cell walls do play an essential role, but the walls themselves are implicitly dealt with. The main advantage of our approach is that the basic model in three dimensions is exactly the same as in two dimensions. Also, the fixed topology that results from the connected cell walls between adjacent cells is an important element of plant tissues. In our model, cells do not roll or slide along each other. At cell division, a cell wall is formed between the two daughter cells, represented in the model by a permanent bond, while bonds with neighbouring cells are redistributed among the two cells, based on the local structure only. The cell walls that are present remain present at all times. So, although the positions of the cell walls are not part of the data structure, their topology is. Any cell wall between two adjacent cells is represented by a single irreversible bond between the particles representing those cells, like in a molecular network. That is the similarity we use, in order to be able to take advantage of the large experience that has been built up with particle models.

The model we have developed is very generic; just a few basic rules are used. The purpose of this paper is a proof of principle of the cell network update algorithm. We present various results of different simulation calculations, for which we use the term sample, to provide this proof. In reality, plants are very complicated organisms, the morphology of which cannot be generated by a very naive model. Plant morphogenesis requires models with agents, or more precisely morphogens, interacting in a complicated manner. What we want to show is that the basic spatial structure on which such models live can be the dynamical network of point particles that we investigate. The more simple that structure, the more efficient such models, especially once agents are included.

Many models used for growing plants actually apply to developing bacterial colonies, or fungal and animal tissue. Byrne & Drasdo [1,2] look at aggregate formation of growing clusters of animal cells that can grow, migrate and divide, and provide an extensive description of the physical background of these mechanisms. Palsson & Othmer [3] use a similar model to describe the growth of slime moulds. The closest resemblance to our model is given by the Voronoi–Delaunay model by Schaller & Meyer-Hermann [4], which provides a systematic procedure of handling growing networks of points in space using Voronoi tessellations. The model has been applied to tumour growth and does allow for cell migration, but could be made to apply to plant cell growth. Very simple models have been developed to study aggregation processes of large numbers of particles, to investigate fractal growth characteristics in asymptotic scaling limits of infinite cluster size. Hatzikirou *et al.* [5] use such a lattice gas cellular automaton model for tumour growth, where the particles move on a lattice. Sozinova *et al.* [6] use a similar model to study bacterial clustering, taking into account the shape of the bacteria. Both models are particle based, and very much simpler than our model, giving extremely fast simulations, but unfortunately they are not applicable to plant tissue. The cellular Potts model (CPM) as developed by Graner & Glazier [7] derives from the classical Potts model in statistical mechanics, developed to describe phenomena in solid-state physics. It treats cells as a collection of points on a regular lattice, and is a widely used and very efficient model to describe a relatively small number of cells, including their dynamical shape and internal structure. More similar to our model is the one developed by Newman [8,9], which describes individual cells as a collection of interaction point particles with pair potentials using the Langevin equations from Brownian dynamics. Like the CPM, it can describe rather detailed dynamics of the cells, and it is similarly restricted in the number of cells it may accommodate. The model of Van Liedekerke *et al.* [10] is aimed at describing mechanical properties of single plant or animal cells, using methods from fluid dynamics. Other models aim more at the cell walls, using similarities between plant cell tissue and soap froths, like the one developed by Corson *et al.* [11]. The VirtualLeaf model as developed by Merks [12] describes the perimeter of plant cells, by a number of points connected by springs, forming the cell wall. Jönsson *et al.* [13] investigate the root tip growth in three dimensions for a restricted geometry in which cells are treated as particles with a polyhedral shape. Barrio *et al.* [14] use Voronoi diagrams in two dimensions, which are essentially equivalent to a particle approach, to study the growth of a root tip. A recent overview of cell-based models is given by Merks [15]. On the other hand, systems of Lindenmayer type [16] are used to model fractal-like growth of whole plants and trees and other larger organisms, from the level of macroscopic subsystems as branched stem parts. The review of Prusinkiewicz & Runions [17] contains both types of models. A more recent review is from Liedekerke *et al.* [18]. For a complete overview of the various models and methods, we refer to these review papers.

## 2. Simulation model

We investigate the dynamics of cells in a model sample of plant tissue. Each cell is identified with just two parameters, its position and its size. The position of cell number *i* is a point, a real vector $r_{i}$ in three dimensions, not restricted to any grid or lattice. Cells sharing a cell wall are connected, and by means of these connections the properties of the cell walls enter the model. The connected cells form a network, which only changes when cells divide and new connections between the old neighbours and the new daughter cells are made. Unconnected cells can never become connected within the model, connected cells always stay connected. The topology of the network changes only due to cell division. Cells interact with each other through a pair potential, generating a pressure. When cells grow, their size parameter $R_{i}$ increases, depending on the local pressure. The simulation of the tissue development occurs in discrete time steps, during which cells can grow and divide. After each step, the system is relaxed towards equilibrium, based on the forces generated by the potentials. Thus, the force serves two purposes. On the one hand, the relaxation of the forces forms an efficient procedure to find the equilibrium configuration, especially after division, on the other hand, as even in the equilibrium state the forces do not relax to zero, they are the source of the pressure.

### 2.1. Cell interactions

The position of cell number *i* is $r_{i}$ and its size is indicated with a parameter $R_{i}$. The interaction potential *U_ij_* between two *connected* cells *i* and *j* is given by

2.1 $$U_{ij}=\frac{1}{2}k{\left(R_{i}+R_{j}-d_{ij}\right)}^{2},$$

where $d_{ij}=\;|r_{i}-r_{j}|$ is the distance between the cells and *k* is a positive constant. This potential is minimal when the distance between the cells just equals the sum of their sizes. When the distance is larger, the potential gives rise to an attraction between the cells, moving them closer together in the equilibration procedure. When the distance is smaller, they are pushed apart. In fact, it is as if connected cells are like point particles connected by a Hookean spring with rest length exactly the sum of the sizes of the cells. Cells that are *not connected* have a potential

2.2 $$U_{ij}=\left\{ \begin{array}{ll} \frac{1}{2}k{\left(R_{i}+R_{j}-d_{ij}\right)}^{2} & \mathrm{if}\;d_{ij}<R_{i}+R_{j}\\ 0 & \mathrm{otherwise}. \end{array}\right.$$

**Figure 1.**
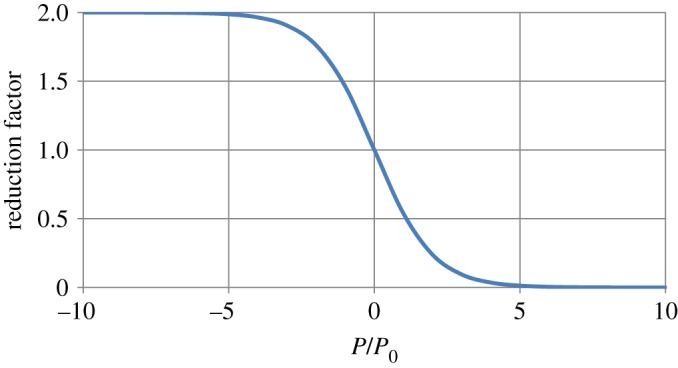
Reduction of the growth rate with increasing pressure (*P*/*P*_0_).

Note that the model can be interpreted as if the cells are spherical. The primary purpose of the potential is for using it in an equilibration procedure. For connected cells in equilibrium, the springs cause the cells to be just touching; for unconnected cells, the springs only serve to remove cell overlap. In fact, the model does not assume any shape of the cells. We come back to this point in figure 1 and the discussion there.

In the equilibration procedure, the force on cell number *i* is derived from the pair potentials with all other cells

2.3 $$F_{i}=\sum_{j}F_{ij}=-\sum_{j}\nabla_{i}U_{ij},$$

where the gradient is taken with respect to position of cell *j*. For most cell pairs the interaction, and hence the interparticle force $F_{ij}$, will be zero. We keep a list of neighbouring pairs, both connected and unconnected, that are within interaction range, or might become so. The list is updated regularly to account for deformation of the tissue sample, as well as after each cell division. Equilibration is performed by repeatedly moving all cells simultaneously in the direction of the force acting upon that individual cell:

2.4 $$r_{i}=r_{i}+\;\delta \;F_{i},$$

with *δ* a model parameter, the old position to the right and the new position to the left. This amounts to a steepest descent minimization of the potential. After a displacement in the direction of the force, new forces are calculated and the cells are moved again. The relaxation procedure is performed with a fixed number *N* of iterations steps of fixed size *δ*.

### 2.2. Cell growth and division

The relaxation procedure does relax the stresses in the network, by changing its configuration as given by the positions of all cells, while maintaining its topology, as given by the connectedness of the cell network. In practice, the fixed topology impedes a full relaxation of the stresses, so there is a remaining stress that influences the behaviour of the growing cells. The pressure $P_{i}$ within cell number *i* after relaxation is calculated using the virial theorem (see for instance [19,20])

2.5 $$P_{i}=-\frac{1}{3V_{i}}\sum_{j}F_{ij}.\;(r_{j}-r_{i}),$$

with the dot indicating the dot product between the force and the distance vector, and

2.6 $$V_{i}=\frac{4}{3}\pi R_{i}^{3}.$$

Note that the pressure is one third of the trace of the pressure tensor.

Next, the cells grow. The growth rate of a cell in our model is affected by the pressure $P_{i}$. The size parameter $R_{i}$ of cell *i* increases with the following:

2.7 $$\Delta R_{i}=R_{i}\;\gamma \;u\;\frac{2}{1+\exp(P_{i}/P_{0})},\;0<u<2,\;\langle u\rangle =1,$$

with *γ* the relative growth rate parameter and *u* a uniformly distributed random variable. If the pressure is zero, the relative increase in size parameter is a random number between zero and 2*γ*, the average relative growth rate then is *γ*. The modification by pressure has a width given by the scaling parameter *P*_0_. If the pressure is high and positive, growth is reduced considerably (figure 1). For negative pressure, the actual relative growth rate is increased compared to the value of the growth parameter *γ*, up to a factor of two for infinite negative pressure. The stochasticity is introduced to simulate real biological systems. Within the model, it can be used to investigate several samples with the same (growth) parameters, but with a different initial random seed, to see what features of the simulated sample are generic for the system parameter values used, and which ones are specific for the individual sample. In that sense, it tests the robustness of the model under certain perturbations. The background of the pressure effect is that real plant cells grow due to turgor. In our model, there is no turgor, but still cells with a high pressure are squeezed in by their neighbours and grow slowly. To describe actual cell growth, the potential can be modified to comply more with actual physical behaviour of cells and cell walls [2].

After the cells have grown, all cells are inspected for division. When the size of a cell reaches twice its original volume, it divides into two daughter cells, each with half the volume of the original mother cell. When a cell divides, the size parameter of the daughter cells become

2.8 $$R_{\mathrm{daughter}}=\sqrt[{3}]{\frac{3V_{\mathrm{daughter}}}{4\pi }}=\sqrt[{3}]{\frac{3V_{\mathrm{mother}}}{8\pi }}=R_{\mathrm{mother}}\;\sqrt[{3}]{\frac{1}{2}}≅0.794\;R_{\mathrm{mother}}.$$

The two new cells are placed at positions

2.9 $$r_{1,2}=r\pm \alpha R_{\mathrm{mother}}\hat{s},$$

where *r* is the position of the dividing cell, and $\hat{s}$ is a unit vector normal to the division plane of the cell. The division plane can be chosen randomly, or depending on the local network, for different submodel choices. We come back to this in §3.3. The parameter *α* controls the initial distance between the divided cells. If *α* is large, the divided cells may strongly overlap with their neighbours; if it is small, they will strongly overlap with each other. In both cases, the forces in the consecutive relaxation procedure can become very large. One of the issues is to find a good choice for *α*. We deal with this issue in §2.4.

**Figure 2.**
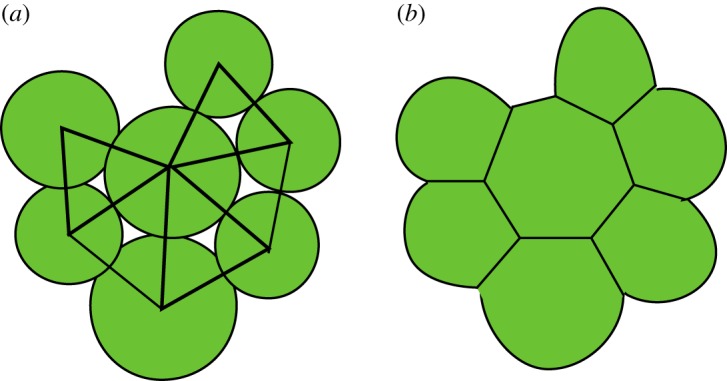
Image of a developed two-dimensional sample with the cells drawn as circles (*a*) and with the cells drawn as polygons (*b*).

The model is developed for three-dimensional structures, so the division vector $\hat{s}$ is a unit vector in three dimensions. By restricting the division vector to the *xy*-plane, any two-dimensional sample will remain a two-dimensional sample, because all forces, and hence all displacements in the relaxation procedure, are also restricted to the base plane. Note that the sample is still three-dimensional, simply all cells lie in the same plane and stay in that same plane, because all movements are also in the plane. At any point during a simulation, a two-dimensional sample could be turned into a three-dimensional one, and one could, for instance, investigate the stability of the two-dimensional configuration. In figure 2, a developed two-dimensional sample is shown. In figure 2*a*, the virtual cells as used in the model are depicted as filled circles with radius that of the size parameter of the cells, connected cells are indicated by a drawn line between their centres. These lines establish the cell network topology. The image suggests that there is intercellular space and that cells can actually overlap, but neither are explicit aspects of the model. In figure 2­*b*, the cells are drawn as filled polygons, without overlap and without intercellular space. The right image can be obtained from the left one by considering the network of connected cells and generating its adjoint network. In the remainder of this paper, we will always use the left type of image, as it gives more information about the simulation result, although the right-hand picture is more reminiscent of actual plant tissue.

After the division stage, the process repeats. Starting from a single cell, the full development process consists of a number of iteration steps, each consisting of three consecutive stages; growth of the cells, possible division of cells and relaxation of the stresses. Note that the value of the growth rate parameter actually sets the time scale of the process.

### 2.3. Cell network update algorithm

The network of connected cells has the cells as the vertices and the distance vectors between the connected cells as the edges. In cell division, the cell walls connecting neighbouring cells remain doing so. In the model, this is no different. We here describe in detail the procedure we have developed for the reassessment of the network connections at division in three-dimensional samples. Exactly the same procedure is used for planar samples. The rules for reconnecting the network are: (i) the two new (daughter) cells are always connected to each other, and (ii) when the old (mother) cell is connected to cell number *j*, the daughter cell nearest to that cell always inherits that connection. To decide whether the other daughter cell may also be connected, we introduce a model parameter *ɛ*; when

2.10 $$\varepsilon <\frac{d_{1j}}{d_{2j}}<\frac{1}{\varepsilon },$$

where $d_{1j}$ and $d_{2j}$ are the distances between the respective daughter cells and cell number *j*, both copies become connected. This triangle criterion is very simple, and does not require detailed inspection of the local network surrounding the dividing cell. However, it may result in too many connections. For further pruning of the network, we use a tetrahedron rule.

**Figure 3.**
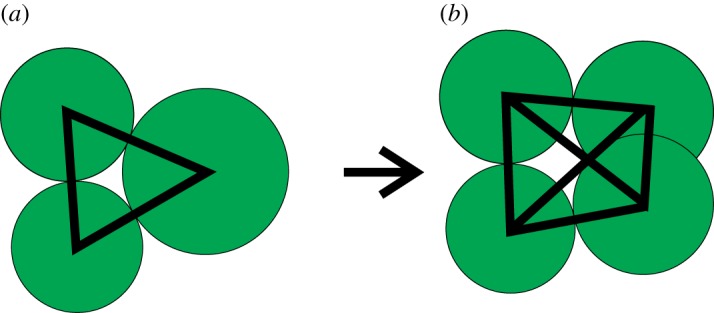
When the big mother cell (*a*) divides into two daughters (*b*) in a planar direction, both daughters cannot stay connected to both other cells.

Consider the small network of three cells as indicated in figure 3, where the largest one divides. The new connections, as generated by the rules we use, then may form a tetrahedron. If the division vector is exactly in the plane of the paper, the diagonally connected cell pairs in reality cannot both share a cell wall, and one must be removed. Whether that is the case for an arbitrary division vector depends on the three-dimensional structure of the tetrahedron. We calculate the minimal distance between points on the connecting line segments between the neighbour cells and the daughter cells to find the thickness of the tetrahedron. If this distance is less than a fixed fraction of the distance between the daughter cells, the longer of the two connections is discarded. For a regular tetrahedron, this fraction is about 0.7. In testing, a fraction of *τ* = 0.4 proved to work well for the tetrahedron rule. The precise implementation of the algorithm we use is given in detail in the electronic supplementary material.

### 2.4. Model parameters

We have performed a substantial number of simulations to find ballpark figures for the various parameters in the model. It appears that there is quite a bandwidth within which the parameters can be chosen to produce samples that can be relevant for actual simulations. Most parameters cannot be varied independently; for instance, a small value of *δ* requires a large value of *N*. Based on these results, we have selected a set of default values that in general works quite well. In table 1, we give the names of the various basic model parameters, their meaning and their default values.

Unless reported otherwise, we have used parameter values as given in table 1. Note that all these parameters are dimensionless. The dimensionless length scale of the model is the size of a cell, of the order of 10 µm. So when using SI units, any length scale in the model is given in multiples of a typical plant cell size, about 10 µm or 10^−5^ m. The time scale in the model is related to the growth rate of the cells. The actual growth rate of cells varies widely; a typical time between divisions is of the order of hours or more, so an appropriate time scale would be of the order of 10^4^ s. The model we use is a quasi-static one; there always is an equilibrium of forces and there are no accelerations. That implies that there is no explicit mass scale that describes how the forces are scaled. The actual force must be derived from comparing the pressure in the model with the actual pressure in cells. For a value of the force constant k=1, the average pressure inside the cells in the model is of the order of unity. The pressure in actual plant cells is of the order of 1 atm, or in SI units 10^5^ Pa. Thus, the force constant in SI units is $k=10^{5}\;\mathrm{Pa}\times 10^{-5}\;m=1\;{\mathrm{Nm}}^{-1}$.

**Table 1.** List of parameters and their default values.

*α*	displacement size at division	0.4
*γ*	relative growth rate	0.01
*δ*	displacement at equilibration	0.2
*ɛ*	double connectedness at division	0.85
*τ*	thickness of tetrahedron	0.4
*k*	force constant	1
*N*	number of iterations at equilibration	10
*P*_0_	width of pressure moderation	1
$\hat{s}$	displacement direction at division	random

For several of these parameters, we will now show how they affect the structure of the simulated tissue sample. For the division vector, we have performed a more elaborate analysis, which is presented in the results section. In the present section, the direction is taken randomly in the plane of the sample. The main purpose of these simulations is to show that the model works well and is able to generate biologically interesting behaviour of the simulated samples.

**Figure 4.**
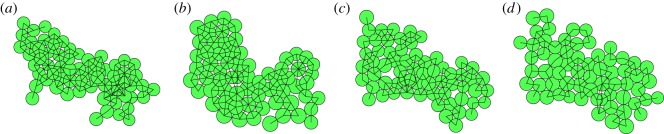
Effect of increasing the parameter *ɛ* from 0.8 (*a*), 0.85 (*b*), 0.90 (*c*) to 0.95 (*d*). For lower values of this parameter, both daughter cells can be connected to the cells to which the mother was connected, for high values only the nearest one is connected. This leads to increasingly tenuous samples for increasing *ɛ*.

In figure 4, we show the effect of the parameter *ɛ*, which determines whether both daughters of a dividing mother cell may be connected, or only one. In all cases, the samples as shown are taken after 150 iteration steps, when the sample contains about 65 cells. The larger the value of *ɛ*, the less likely it is that both daughters remain connected and consequently the samples are increasingly tenuous in structure, tree-like with thin branches and many forks. For low values in many cases both daughters are connected, and a very much connected lump-like structure is formed. Sensible values of *ɛ* are between 0.75 and 0.95. For the remainder of this study, we have chosen an intermediate value of 0.85.

The displacement size, parameter *α*, does not have a direct effect on the structure of the sample. Large values of *α*, of about unity, make that the two daughters are fully separated, but give substantial overlap with the neighbouring cells. Small values of *α* avoid overlap with neighbours, but lead to a large overlap between the daughter cells. In both cases, relaxation becomes cumbersome, needing many steps or leading to unstable runs. We have opted for an intermediate value of α=0.4.

The Hookean spring force in molecular and colloidal settings is considered a soft potential, allowing for considerable overlap between particles. It works well though to keep bonded particles together. Note that the parameter *δ* in equation (2.4) has a dimension, its size is related to interaction constant *k*. In fact, a system with a spring extension of *r*, and hence a spring force −kr, would be reduced to its rest length in a single step by moving both ends of the spring with δ=1/2k. For *k* = 1 thus a value of δ=0.5 would seem to be the most efficient choice. In the simulations, there often are many overlapping neighbours for a single cell, and a displacement according to the combined forces of all neighbours can easily lead to a severe overshoot of the equilibration procedure. A smaller value of *δ* avoids that, but only leads to a partial relaxation, so the procedure is repeated. A fixed value of δ=0.1, combined with a number of *N* = 10 iterations and a force constant *k* = 1 has been used, unless reported otherwise.

**Figure 5.**
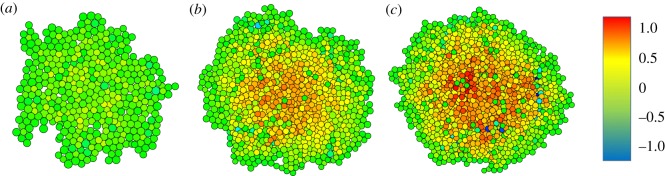
Effect of the parameter *P*_0_. The scale of the pressure modification of the growth is 0.1 (*a*), unity (*b*) and 10 (*c*). The colour of the cells is an indication of the internal pressure, blue is minus unity, green is zero, red is unity. For low values of *P*_0_, the growth is restricted, there are few divisions, resulting in a small sample with homogeneous pressure. For large values, cells grow and divide freely, giving a large sample with a high central pressure. The parameter has little effect on the structure, but a large effect on pressure profile and size.

The pressure parameter *P*_0_ determines how much the growth is affected by the pressure *P_i_* of a cell. A high positive value of the pressure gives a much reduced growth rate, a high negative pressure an increased rate, with *P*_0_ setting the scale. For small values of *P*_0_, cell growth is substantially reduced, leading to few divisions and much relaxation of the pressure. High pressure only occurs at cells that have recently divided. In figure 5*a*, we used *P*_0_ = 0.1. After 250 time steps, a single cell has grown into a sample of 420 cells. The colour indicates the pressure according to a standard heat map colouring (blue–green–yellow–red), with blue a pressure of minus one, green zero and red plus one. Cells with a pressure below unity are coloured blue, cells with a pressure above unity are coloured red. Both occurrences are rare. The highest pressure figure 5*a* is 0.3 (yellow–green colour), the lowest just below zero (green). All in all, the pressure distribution is quite homogeneous. For *P*_0_ = 10, figure 5*c*, red cells occupy the centre of the sample, while low pressure cells are seen closer to the perimeter. Here the highest pressure is just above unity, the lowest just below minus unity. The sample contains 1343 cells, grown in the same 250 time steps. The default value is *P*_0_ = 1, figure 5*b*, which contains 1042 cells. The highest pressure is 0.8, the lowest −0.6.

### 2.5. Equipment

The plant simulations are performed using private code that was developed for molecular and colloidal simulation, modified to include the specifics we mention, but not intended as a package with a user-friendly interface for use by biologists. As stated, the purpose of the present investigation is to show the viability of the approach. All simulation results presented in this paper are obtained on a HP Z400 workstation with a 2.66 GHz Intel^®^ Xeon quad core processor, using Fortran code on just a single core, compiled with a Compac Visual Fortran 6.5 compiler. The full code is available in the supplementary material.

**Figure 6.**
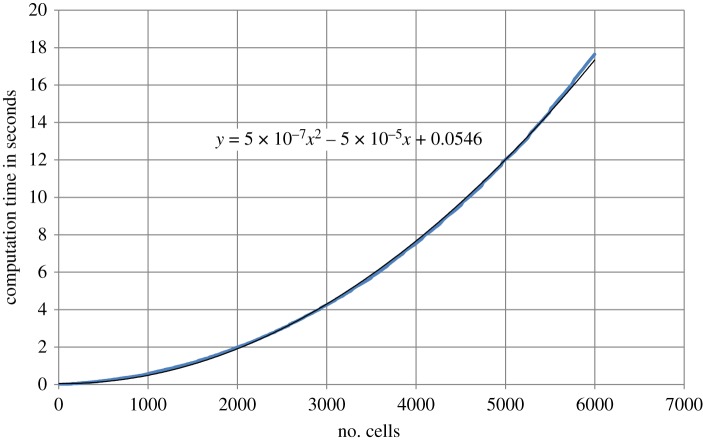
Computation time in seconds (blue line) for generating a cluster of *N*_c_ cells as a function of *N*_c_. The quadratic fit (black line) is consistent with an effort increasing roughly linearly with the number of cells.

Most runs complete in a matter of seconds. In figure 6, we have plotted the time it takes to produce a cluster of *N*_c_ cells as a function of *N*_c_. Because the cluster grows, the number of computations per time step increases with cluster size. In creating a cluster of size *N*_c_, the total effort accumulates, mathematically it is integrated. If the calculational effort of a single step of the code increases linearly with *N*_c_, the time it takes to create a cluster of size *N*_c_ will be proportional to *N*_c_^2^, which is what we observe, the data fit a quadratic trend line (figure 6). More important is that the part that codes for the division only investigates the connected neighbours of a dividing cell. Once the full cluster is sufficiently large, this number of connections (adjacent cells with which a cell wall is shared) will on average become constant. Hence, the computational effort per cell division on average becomes constant. The total amount of effort spent on division in producing a cluster of size *N*_c_ hence increases linearly with *N*_c_ for larger clusters. The pair interaction, in principle, increases quadratically, because the number of pairs of cells increases quadratically, but because only adjacent cells interact, the effort can also become linear. For moderately large clusters as we have investigated, the bookkeeping involved in neighbour list maintenance, which is used in our model, has a minor contribution to the overall effort. Current I7 processors are slightly faster than the one we used and use of more cores can further increase speed, so the absolute effort as reported is a conservative indication.

## 3. Modelling results

### 3.1. Fractal-like growth

We start with the basic model, as described above, using the default parameter values from table 1. Division vectors are restricted to the *xy*-plane, leading to a planar sample, which can easily be inspected visually. The default growth rate of γ=0.01 is rather high. In each step, the radius of a cell increases by 1%, so its volume increases by 3%, and if it would keep doing so, the volume would double in about 23 steps, after which the cell is up for division. We observe that in 250 iteration steps the sample grows from a single cell to about 1000 cells (figure 5*b*). If all cells would have grown steadily at the set average rate, there would have been $2^{250/23}\approx 2000$ cells, so the remaining pressure after relaxation does impede growth. Still, in many steps there is more than a single cell that divides. Owing to the pressure impeding growth, the sample mainly grows on the perimeter, and it appears to do so quite freely.

**Figure 7.**
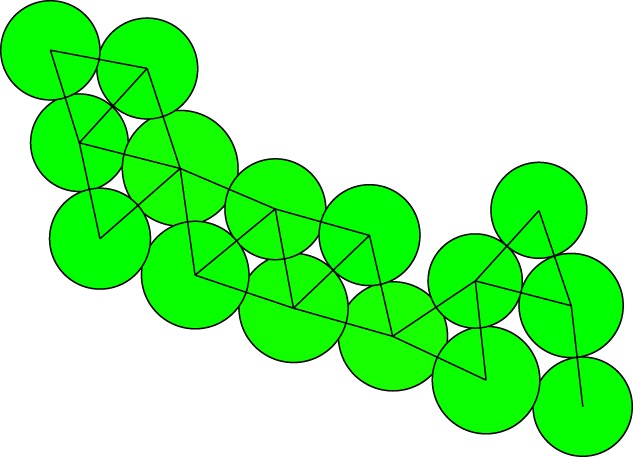
Sample after 100 iterations. Cells are visualized as circles with radius that of the size parameter *R*, lines indicate connected cells.

**Figure 8.**
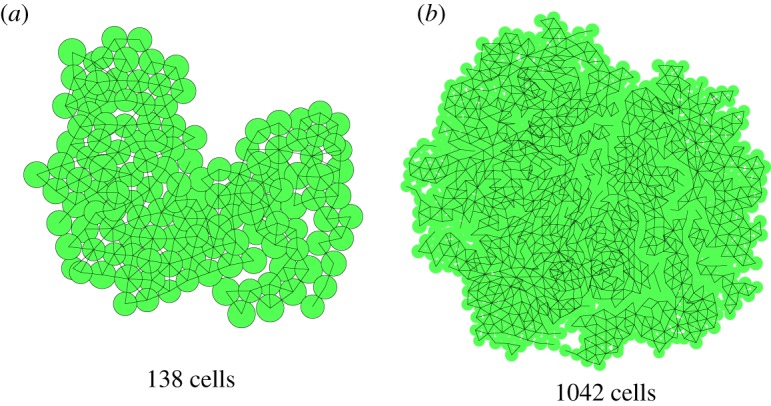
The same sample after 175 (*a*) and 250 steps (*b*), both grown from a single cell. These images are taken from a single run, continued from the previous image. In (*b*), the perimeter of the circle representing the cell is left out, making the network of the cells stand out more clearly. The network has many branches and peninsula-like structures, giving it a fractal-like appearance.

After 100 iteration steps, our sample looks as the one in figure 7. The network of 15 cells is fully connected, for a large part in a quite regular way, forming a slightly distorted triangular lattice of bonds. Figure 8 shows the same sample after 175 steps, now containing 138 cells, and after 250 steps with 1042 cells. For clarity in the largest cluster, the circles representing the cells are left out; only the links of the network drawn. The linked cells still form a (distorted) triangular network, while the overall structure looks like a sort of random snowflake, with a fractal-like appearance. Such branched structures do occur in reality, but are not very common. We will now investigate whether modified growth rules, not depending on agents, can affect the structure.

### 3.2. Pressure distribution

**Figure 9.**
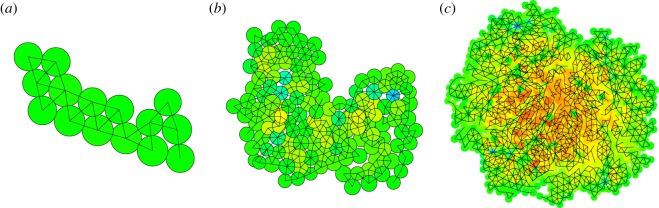
Pressure distribution. These images are the same as in the previous two figures, grown from a single cell in (*a*) 100, (*b*) 175 and (*c*) 250 steps. The colour shows the pressure distribution within the sample, where blue indicates a pressure of minus unity (negative), green zero pressure and red a pressure of unity (positive). For intermediate pressures, a heat map rendering is used.

In figure 9, the exact same samples are rendered, we now focus on the internal pressure. In the smallest sample (figure 9*a*), all cells have a pressure close to zero (green), their growth rate is not affected very much according to equation (2.7). For the sample after 175 iteration steps (figure 9*b*), there are several blue cells. Most of these have seven or even more linked neighbours, and are not filling the space they have available. Hence, the pressure is negative, the growth rate is increased, and many are close to division. There are a few yellow cells, which have a pressure of about 0.5. They have a decreased growth rate. Cells on the perimeter of the cluster have zero pressure, which means they will grow at about the average rate set by the parameter *γ*. For the cluster in figure 9*c*, after 250 steps, the blue cells occur mainly near the edge of the structure, while the centre of the cluster contains many red cells, with high pressure, growing slowly. We see how the pressure effect affects the structure of the sample. We investigate the effect of further restrictions.

### 3.3. Choice of the division plane

**Figure 10.**
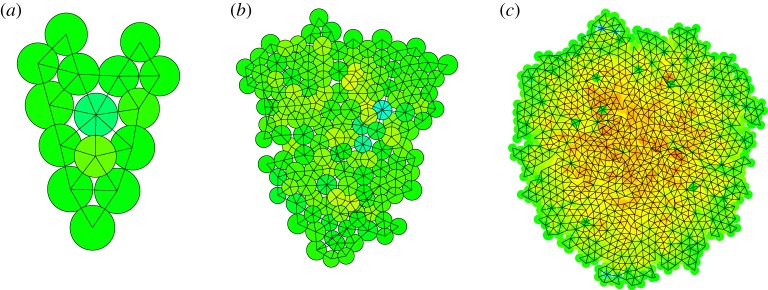
Sample with the orthogonal option switched on, after (*a*) 100, (*b*) 175 and (*c*) 250 steps. The choice of the division plane is the only difference between this and the previous sample. The structure is much more regular, with less deep invaginations, but still branches develop.

In the samples shown so far, the normal vector to the division plane is chosen randomly, and consequently so is the division plane itself. It is the same vector as the displacement vector of the daughter cells at division, the vector $\hat{s}$ in equation (2.9). This turns out to be a major factor in the randomness as observed in the larger clusters. In figure 10, we show clusters obtained by choosing the vector orthogonal to the link to the closest connected cell. The effect is that both daughter cells are connected to that particular neighbour, thus enhancing the connectivity of the network and reducing the possibility of long dangling strands. The sample structure is much less fractal than that of the one presented in figure 8. Clusters tend to be more regular and form a cell network with, for a large part, almost equilateral triangles. We have not investigated the effect of the choice of the division plane for three-dimensional samples on their structure, but we did use a division vector perpendicular to the nearest connected neighbour in generating the samples. A biological motivation for this option is that the division plane in biological cells is often perpendicular to the longest axis of the cell. In our simulation, the nearest neighbour in the network does specify the wanted direction.

### 3.4. Three-dimensional samples

**Figure 11.**
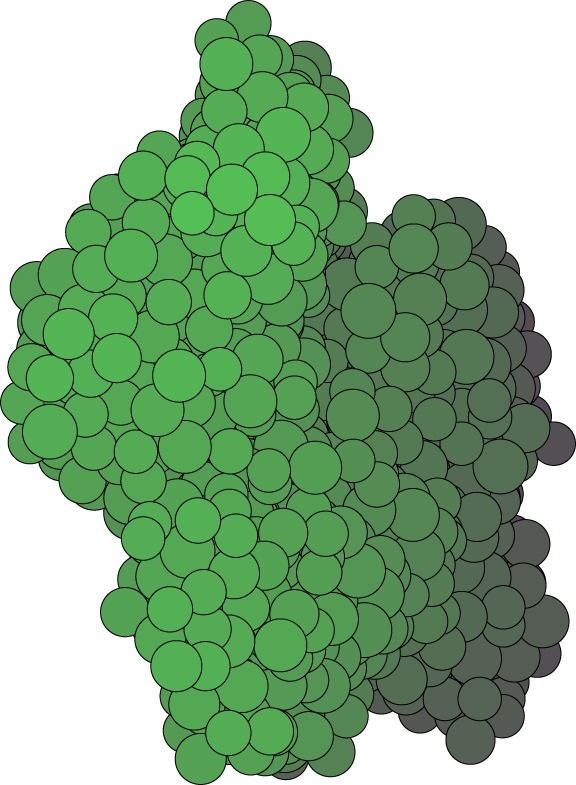
Three-dimensional sample at the default parameter values, grown in 250 steps from a single cell to 1314 cells. Shading is used here as a depth cue, the darker the colour of the cell, the further back its position in the sample.

A sample generated with the same parameter values as for the two-dimensional samples described above, with the division vector $\hat{s}$ (normal to the division plane) of the dividing cells perpendicular to the nearest neighbour link, is shown in figure 11. After 250 steps, it contains 1314 particles. Shading is used to indicate the position of the cell with respect to the plane of the image; brightly coloured cells are to the front of the image plane, darker shaded ones to the back. A movie of the growing cluster is available in the electronic supplementary material, as well as one of the sample being rotated. Our model is able to handle three-dimensional samples equally efficiently as two-dimensional ones; planar samples are just a special case.

## 4. Discussion

The results presented in this paper depend on the default parameter values used and the specific random seed chosen for the run. The results are nonetheless quite generic. We have repeated the simulation with the same parameter values at other random seeds and obtained similar results (see electronic supplementary material). The main purpose of the paper is to show the feasibility of our approach and give a proof of principle of the network update algorithm at cell division. After each division, the model has to determine anew the fixed network of cells bonded by their cell walls, and it appears that this can be achieved by including just a local computation of the network rearrangements at division, with a single algorithm that applies in both two-dimensional and three-dimensional cases. The computational effort involved in this scales linearly with the number of divisions, and hence the number of cells. For the simple growth dynamics in the current model, in fact the division algorithm takes up a substantial part of the effort. Once the growth is described by agents, and their transport over the network, it is to be expected that this transport will become the dominant factor.

Many of the samples presented show deep invaginations. Smaller samples, specifically when using the orthogonal division rule, show a smooth perimeter, but when sample size increases, the invaginations start to grow. Most real plant samples have a smoother perimeter than we obtain in our simulations. The reason probably is that, in real plants, growth and division is controlled by agents, which may involve mechanisms that restrict the spontaneous development of branched structures. The branched morphologies we obtain could be typical for tumours.

A typical phenomenon we sometimes obtained is the occurrence of holes, parts where the bond network does not cover the interior of the sample. In the two-dimensional samples, the holes are well-defined parts of the topological structure, in three-dimensional samples there are internal parts of the structure that have little or no bonds. It can, in principle, be investigated by looking at whether the network is fully built up of triangles (in two dimensions) or tetrahedrons (in three dimensions), or that elements with more edges occur. The occurrence of such elements in the current model is the result of the removal of bonds. A removal procedure based completely on this triangulation or tetrahedralization has been considered, but we have not been able to find one that leads to a local calculation only. However, holes are quite rare in our samples, and often occur only at the larger sample sizes. Their occurrence can be restricted by using other values for the parameters in the division algorithm, but we have not been able to fully avoid them.

We have shown that our model is able to handle tissue samples of up to thousands of particles quite efficiently. The code we have developed has not been optimized for parallel processing, so we have only tested it on a single core of a quad core processor. In general, molecular dynamics types of simulations are well suited for parallelization, so one may expect the efficiency of the code to increase almost linearly with the number of cores. That would mean that on a standard quad core PC the full growth of a sample of the order of 2500 particles can be performed in the order of one second computation time.

External or internal effects may influence the characteristics of the cell wall, and hence the individual growth parameters of the cell [21]. The growth rate modification due to the internal pressure is such an example. In our present model, we have assumed the growth is not restricted by resources, nor is it modified by agents. In a further developed version of the model, agents and resources will be included, which are transported either actively or passively between connected cells. The concentration of such an agent, for instance auxin, then determines the individual growth behaviour and other model parameters that have been introduced above. Differentiation of cell growth parameters between cells, based on internal agents or external influences has no effect on the overall performance of the model. In fact, the model is just a vehicle for this agent-based model, to generate the network on which the agents diffuse. In practice, the agents are mainly responsible for the generated morphology. Diffusion of agents is likely to take an important part of the computing effort in an extended model, but compared to models that use internal cell gradients it will still be quite efficient. Of course, if those cell gradients are essential for the biological system, more complicated models cannot be avoided.

The isotropic interaction between connected neighbouring cells can be interpreted as indicative for spherically symmetric cells. Stresses within the sample lead to deviations from this spherical symmetry. A rather straightforward way to introduce such deviations explicitly into the model would be the use of anisotropic interaction potentials, and use of the full pressure tensor. Introduction of such anisotropy in colloidal systems has shown to be a viable option, though it results in more complicated code [22]. A much simpler approach could be to use a dumb-bell model for slightly non-spherical cells, with two connected point particles representing a single biological cell, without a cell wall separating the two. Such a modification could be introduced without too much effort and without affecting the simplicity and efficiency of the current code. Indeed, it would be possible to deal with cells of any shape by having a single cell described by any number of points, in which case our model would become rather similar to the one as developed by Newman [8].

The effect of the division plane on the structure of the cells network in the generated tissue, as we have investigated in this study, has been the subject of explicit study elsewhere. Sahlin and Jönsson [23] have shown that different rules for two-dimensional epithelian cell growth can explain observed tissue patterns in *Arabidopsis thaliana*. One of the earliest accounts of cells dividing along their geometrical long axis is by Hofmeister [24]. Gibson *et al.* [25] have demonstrated that in a simple geometrical model for polygonal cells the surrounding cell topology is a key element in this. In fact, in our model the same effect is observed; moreover, our observation is based directly on the surrounding cell topology.

## Supplementary Material

2Dmovie

## Supplementary Material

3Dmovie

## Supplementary Material

3Drotate

## Supplementary Material

Code

## Supplementary Material

Various samples

## Data accessibility

There are no data. The full fortran code that has been used for the simulation is available in the electronic supplementary material (file code.zip).
