# Supplementary material for: Algorithm for a particle-based growth model for plant tissues
Source: R Soc Open Sci. 2018 Nov 28;5(11):181127. doi: 10.1098/rsos.181127 (PMC6281936; doi:10.1098/rsos.181127)

In the following we have used different seeds in the random growth rate term, leading to different “samples” at the same parameter values. The first are the results as shown in the paper, the four alternative results presented here consistently use four different random seeds.

Default parameter values. Single cell in 150 steps. Compare Fig 4

Eps=0.80


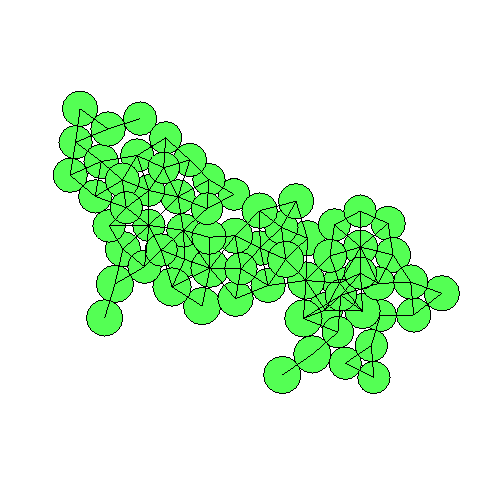

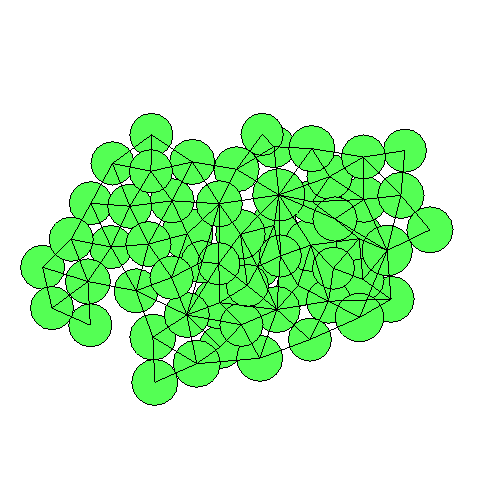

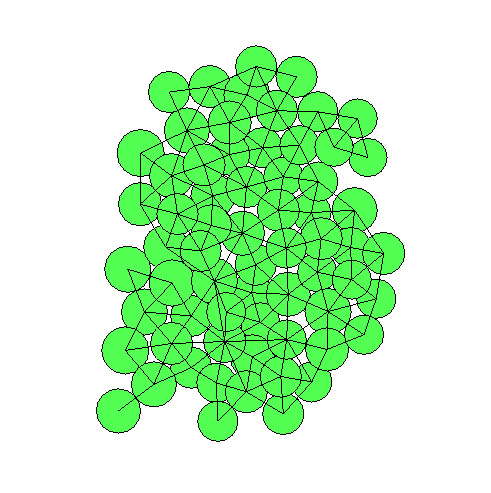

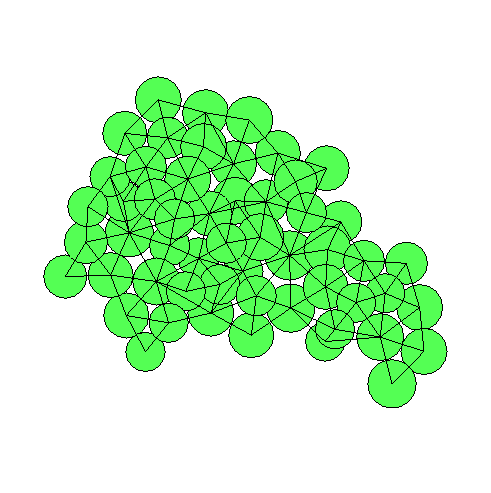

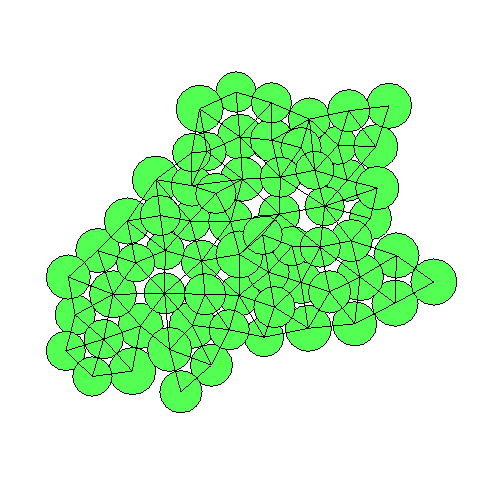


Eps=0.85


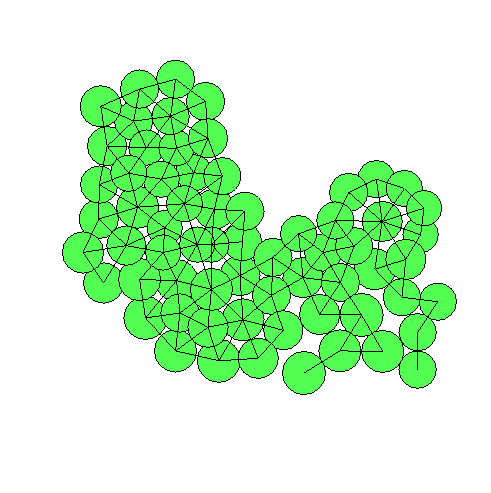

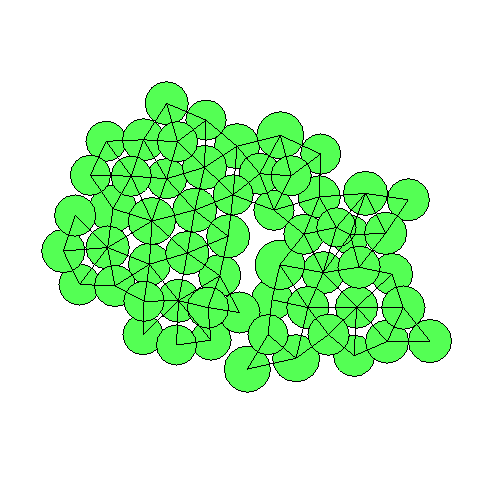

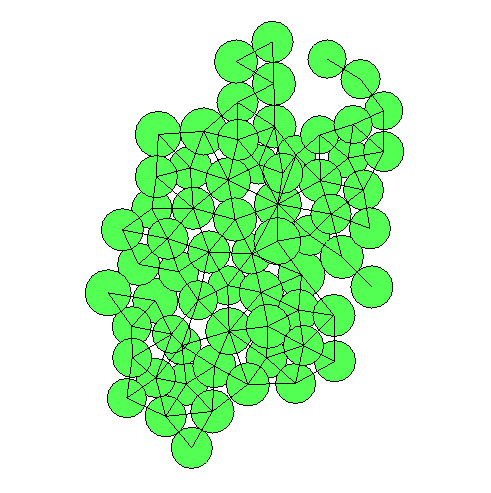

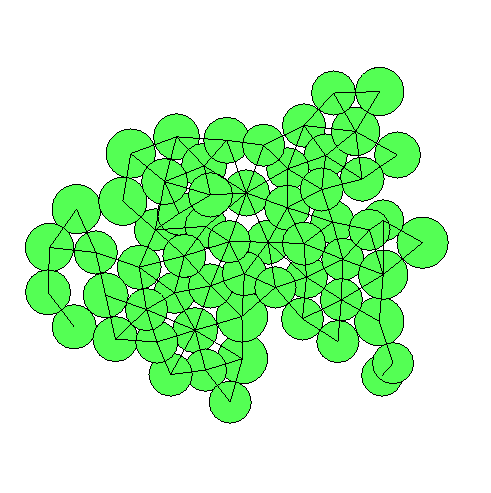

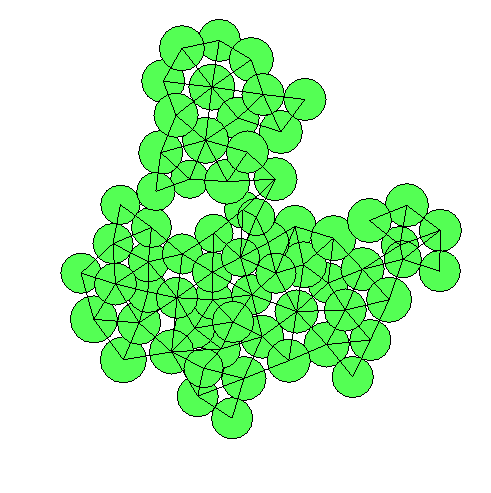


Eps=0.90


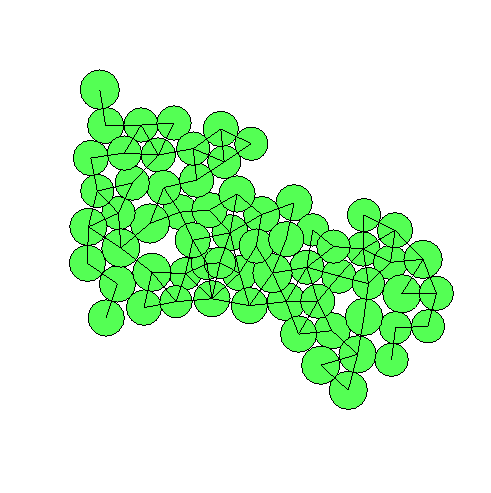

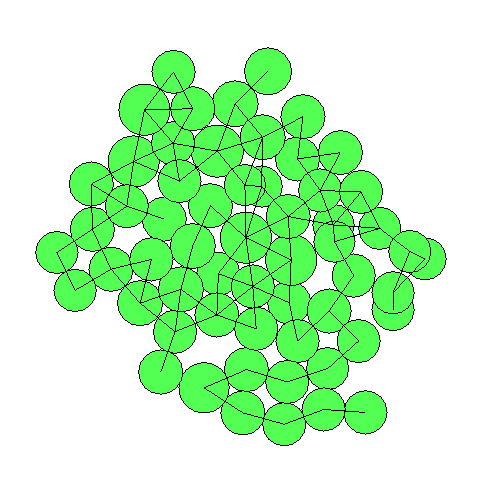

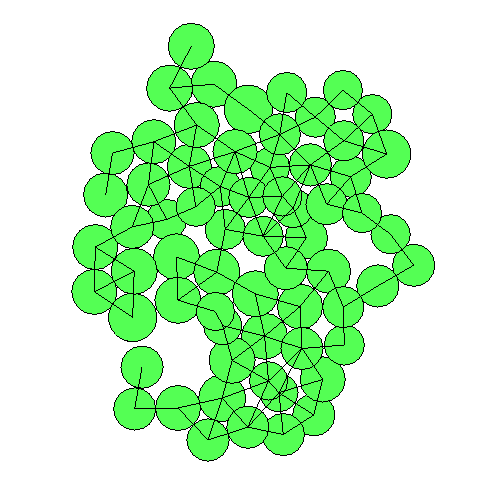

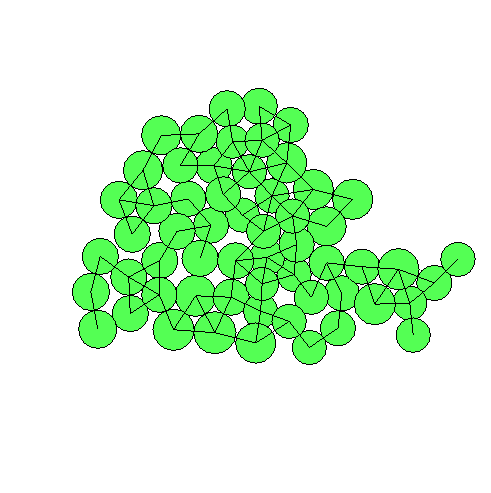

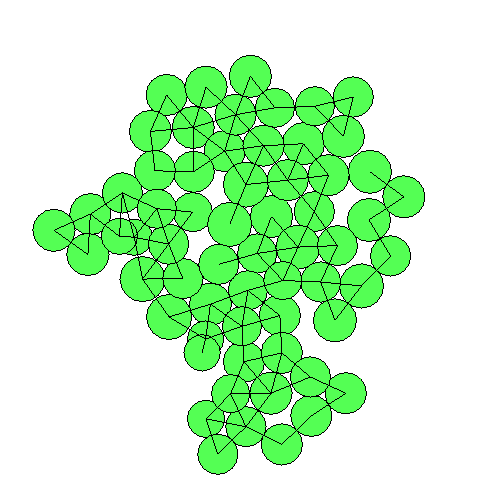


Eps=0.95


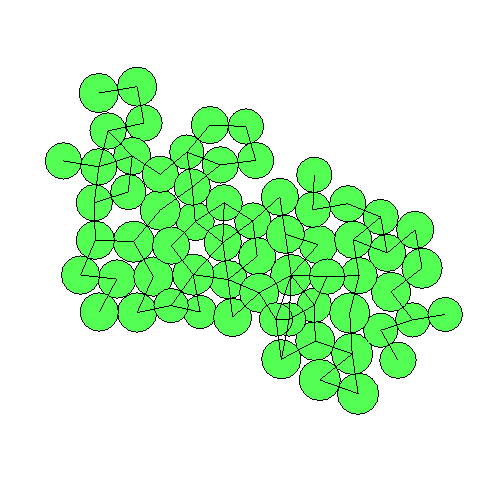

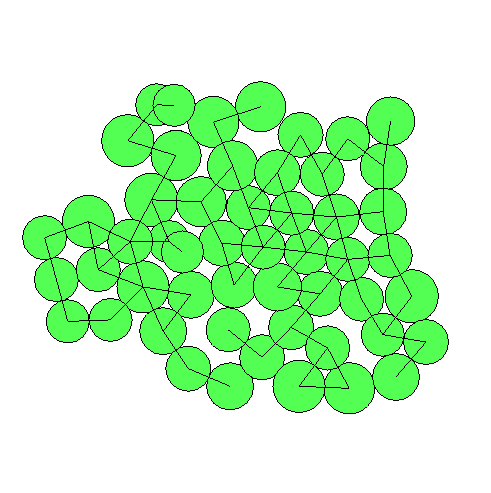

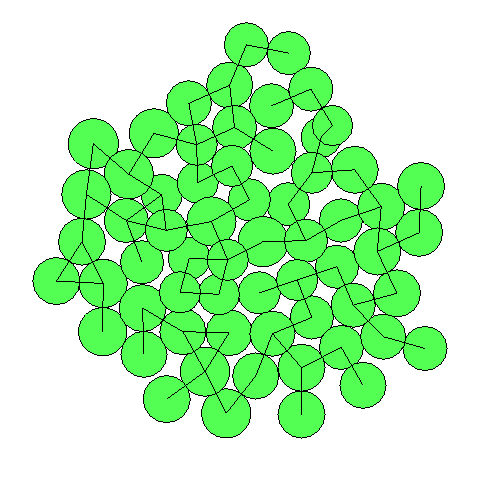

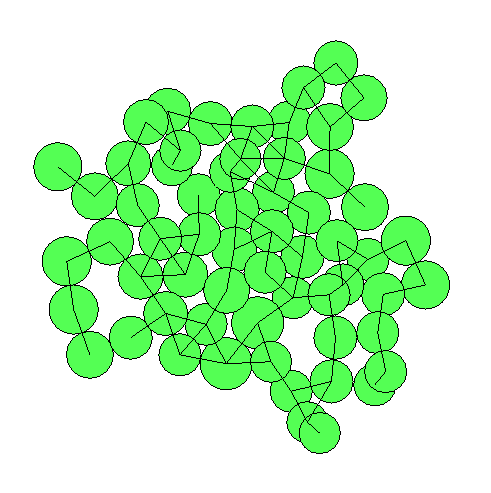

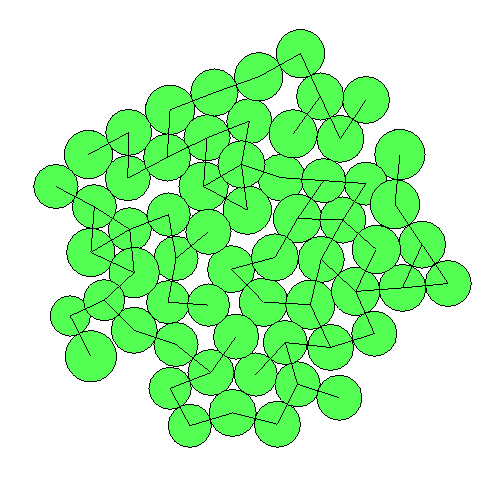


Pressure threshold value. Single cell, 250 steps, default parameter values. Compare Fig 5

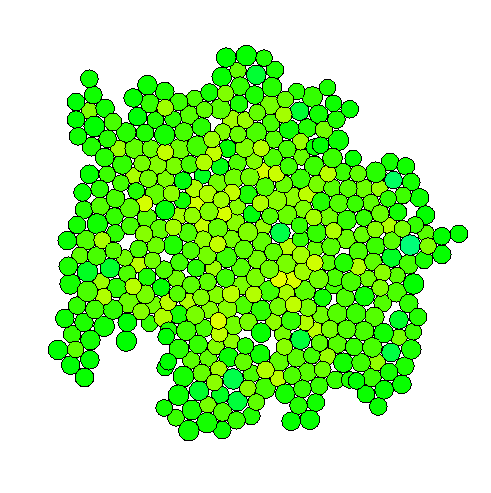

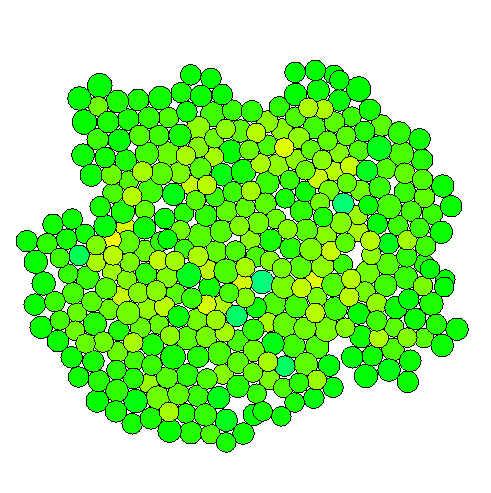

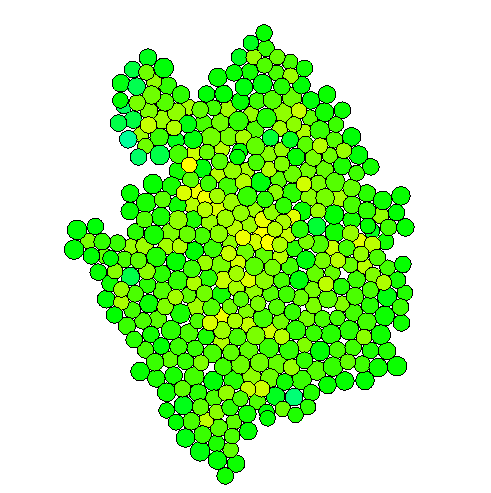

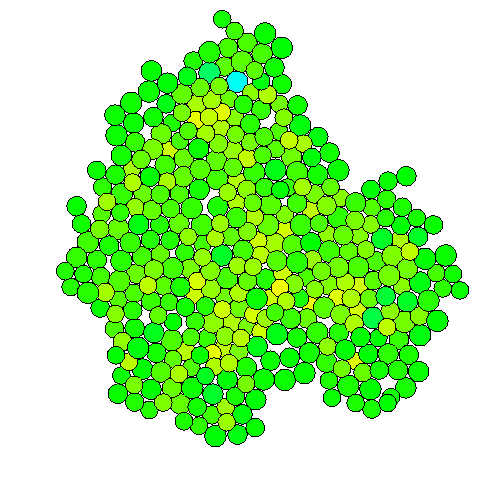

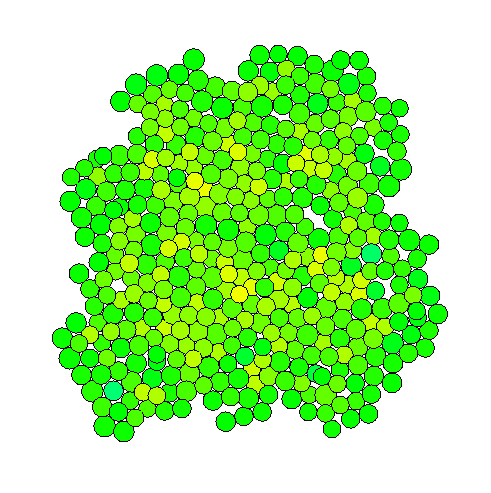

$$P_{0}=1$$

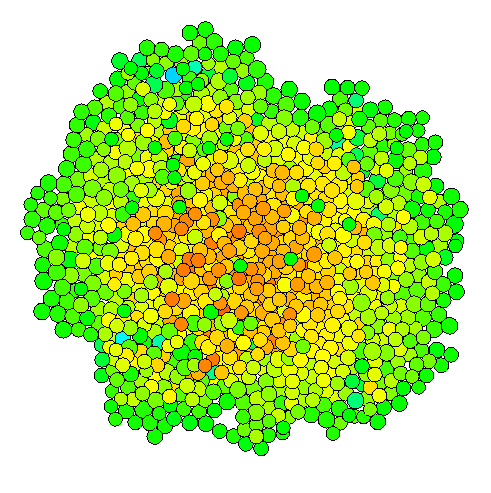

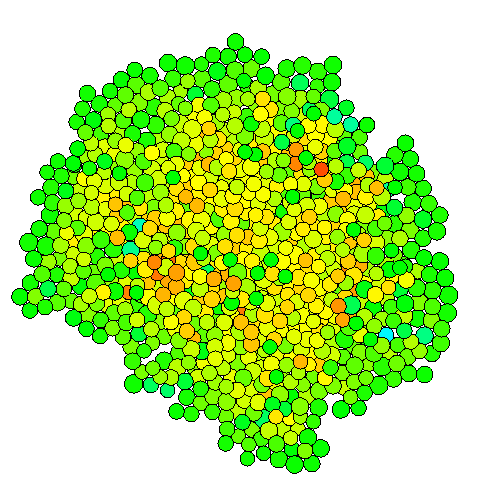

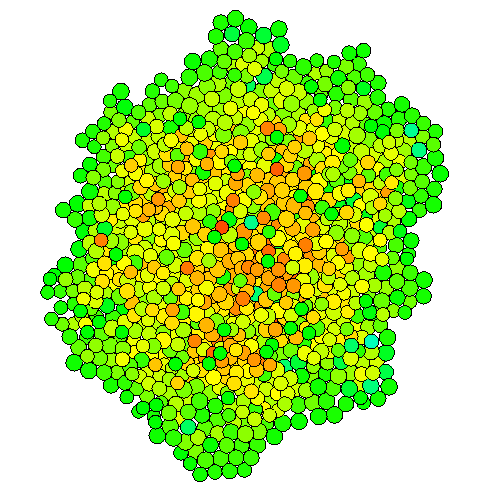

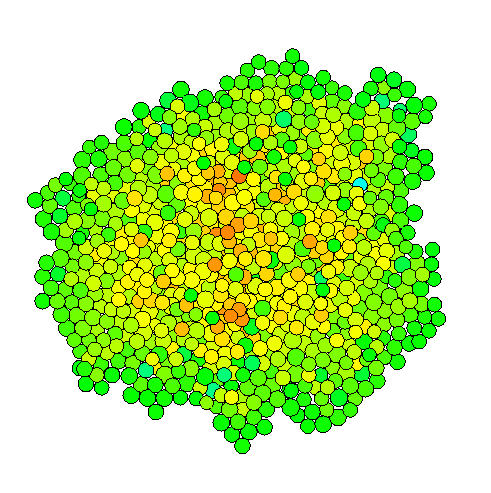

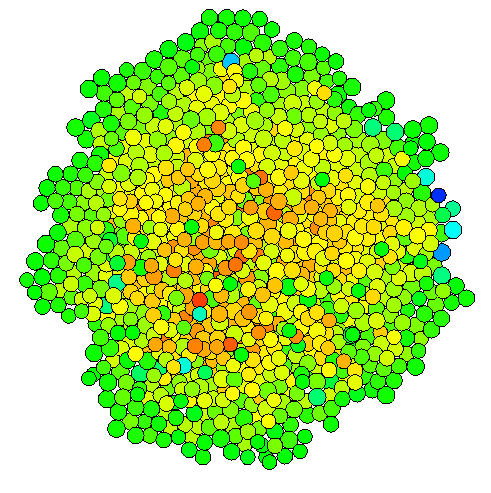

$$P_{0}=10$$

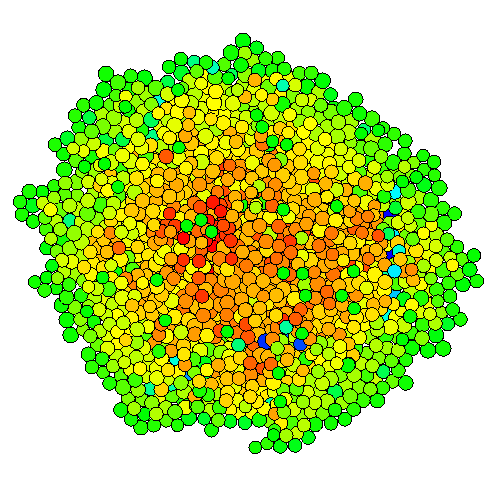

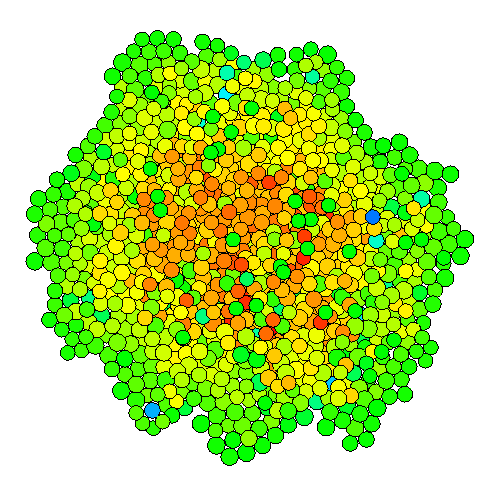

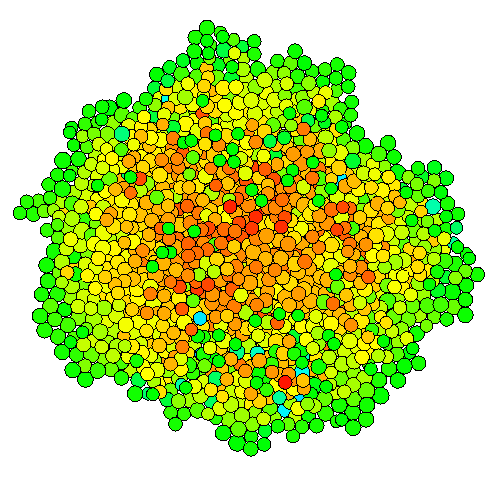

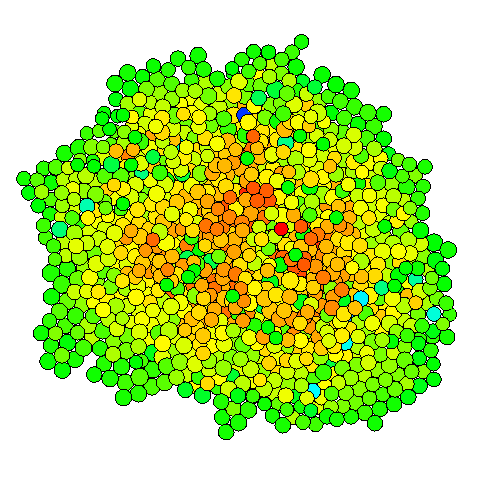

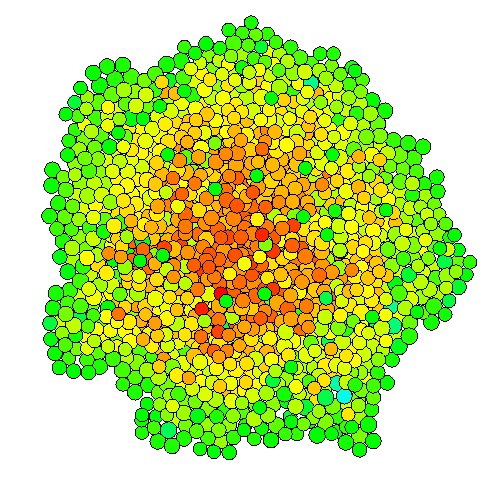


Default parameter values. Sample development in time. Compare Figures 7,8 and 9

100 steps


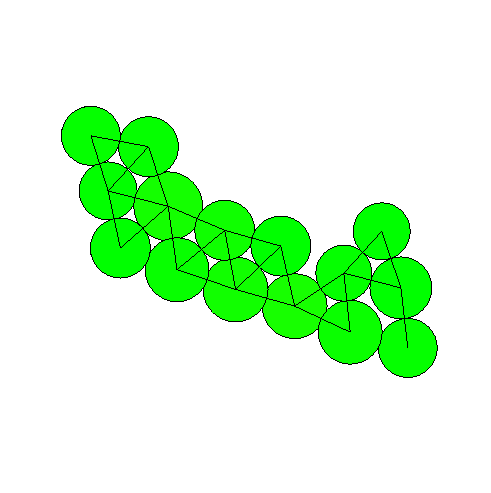

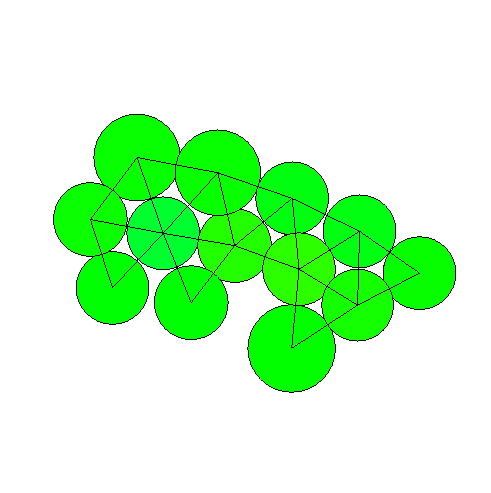

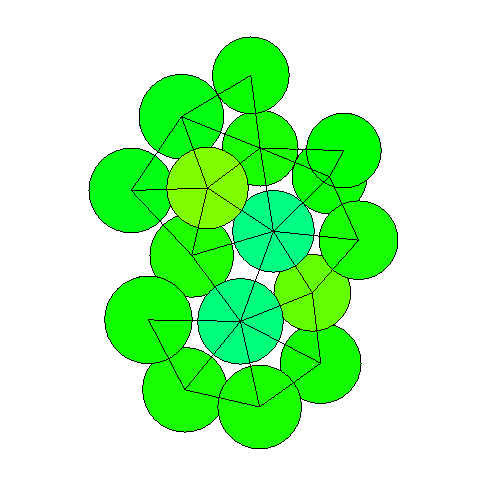

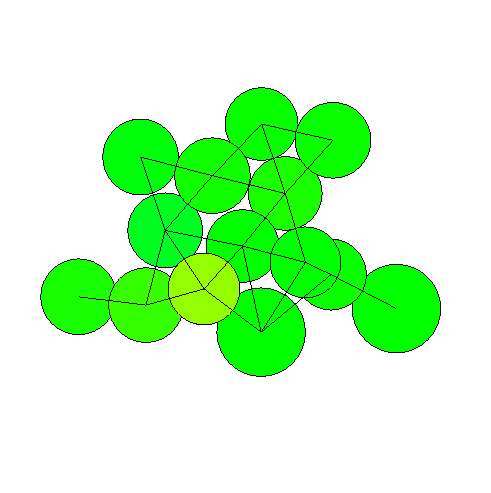

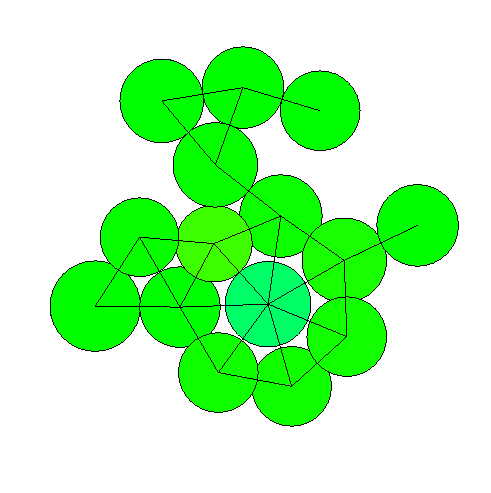


175 steps


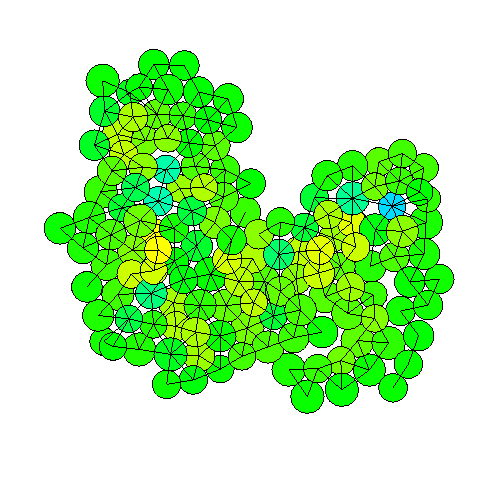

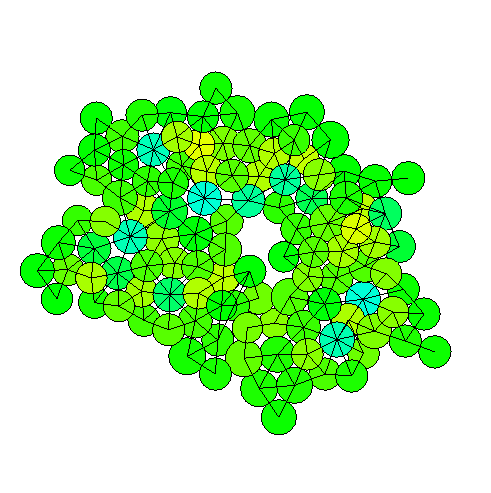

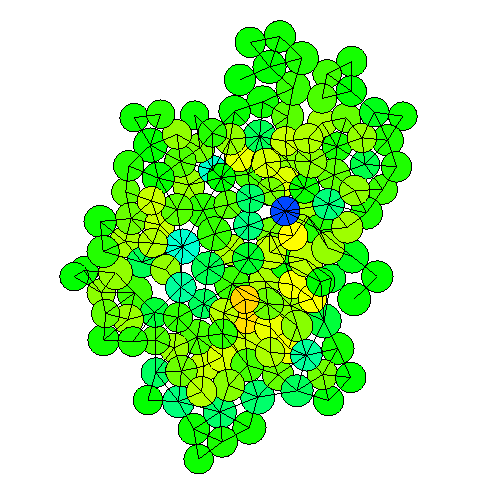

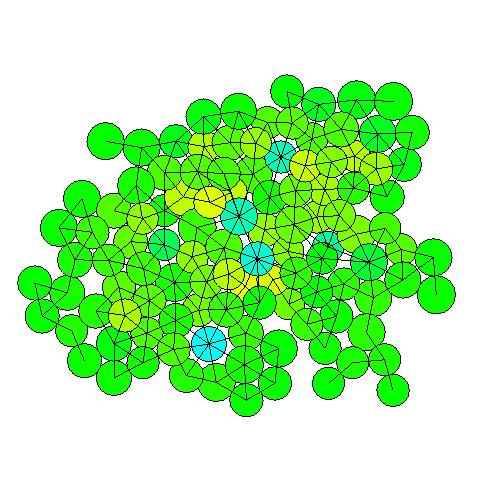

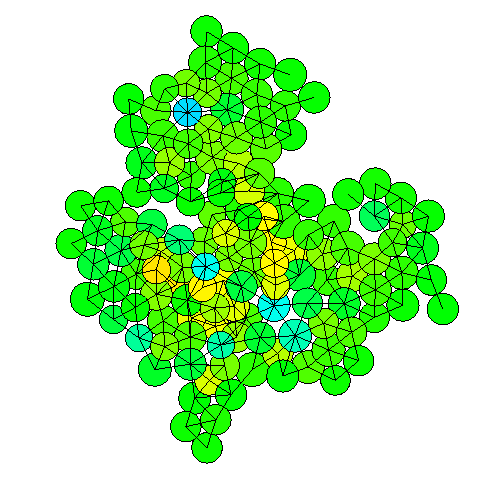


250 steps


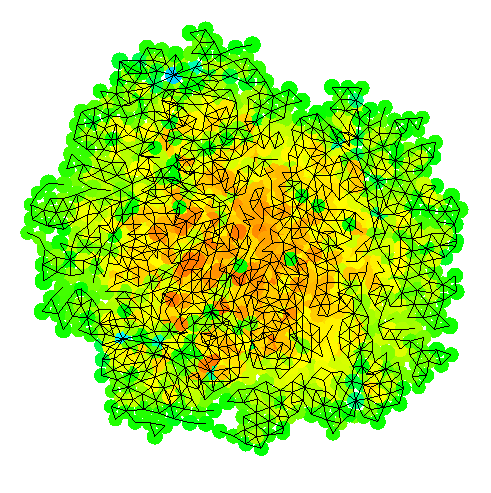

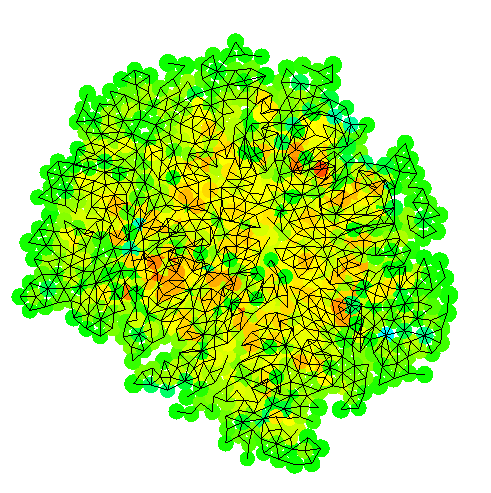

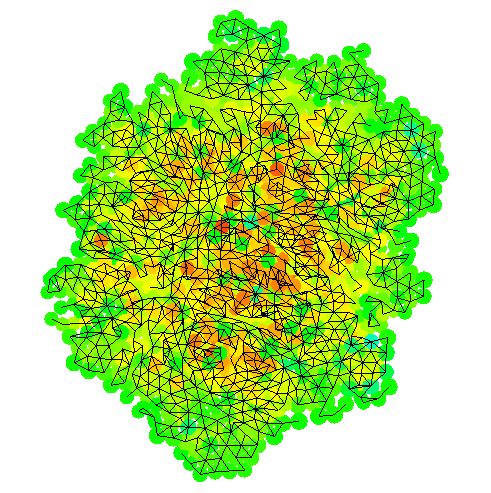

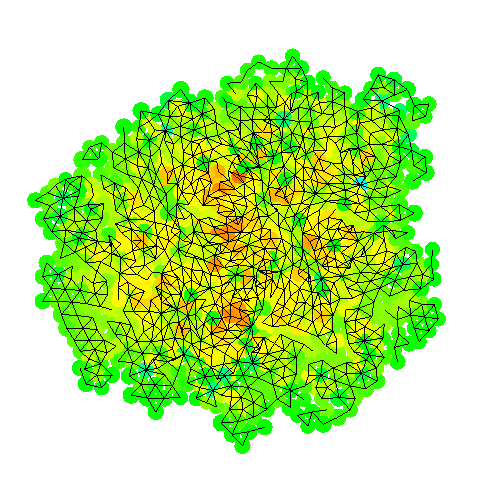

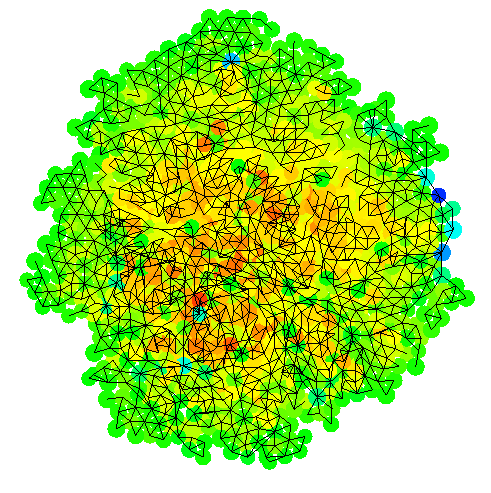


Orthogonal option for the division plane, compare fig. 10

100


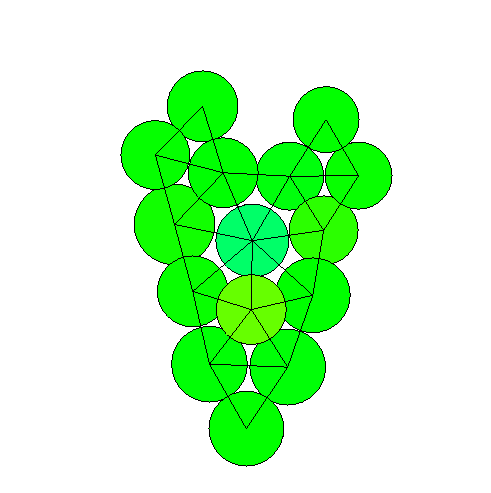

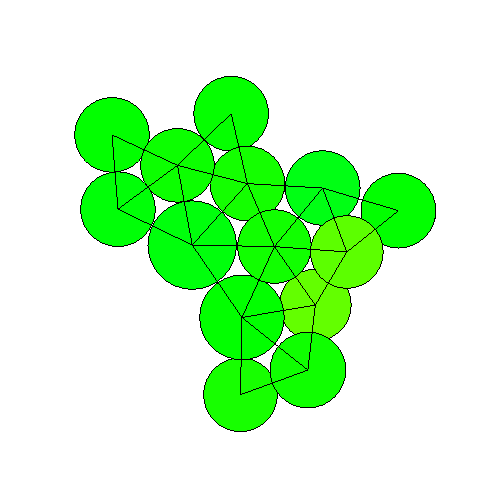

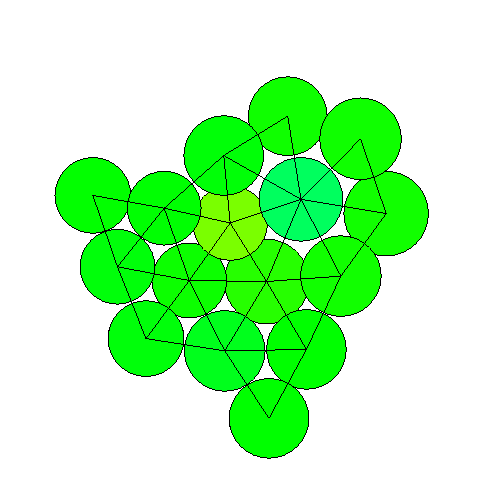

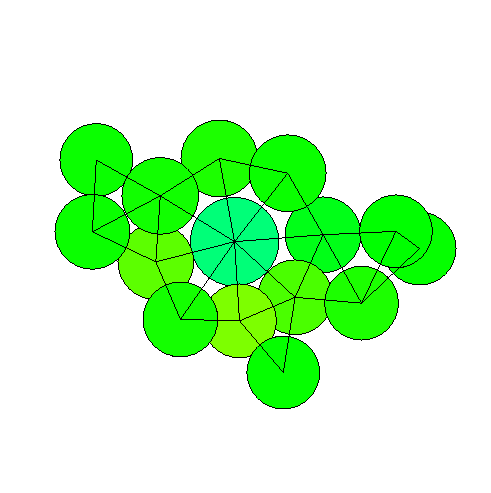

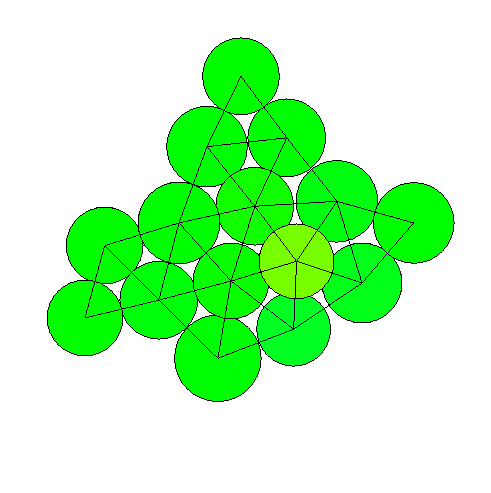


175


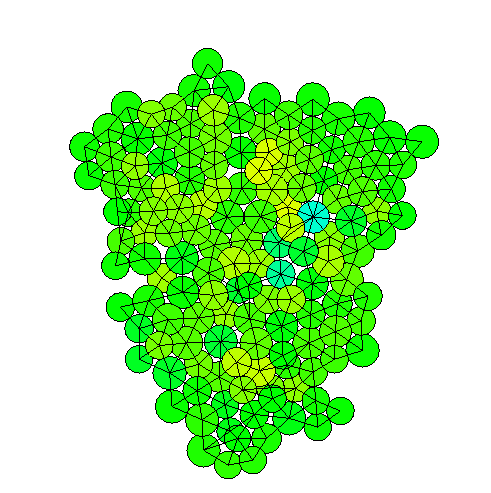

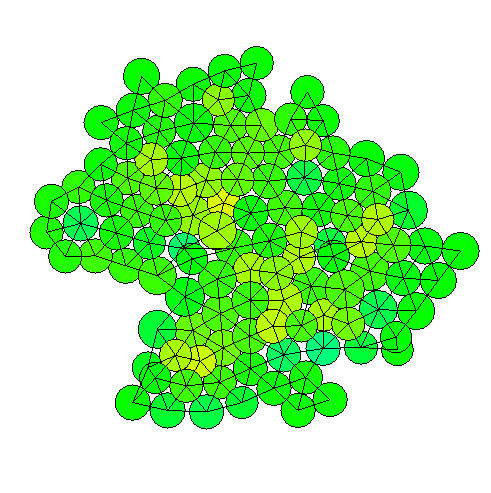

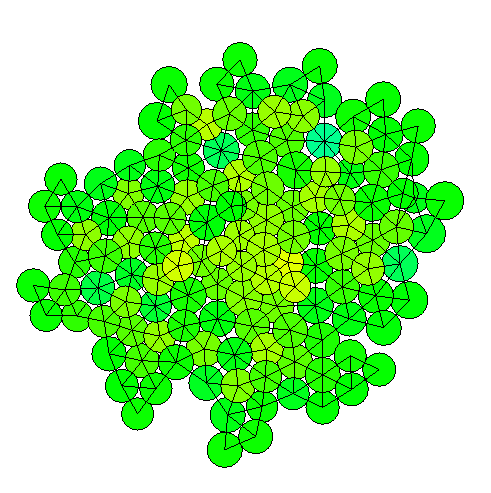

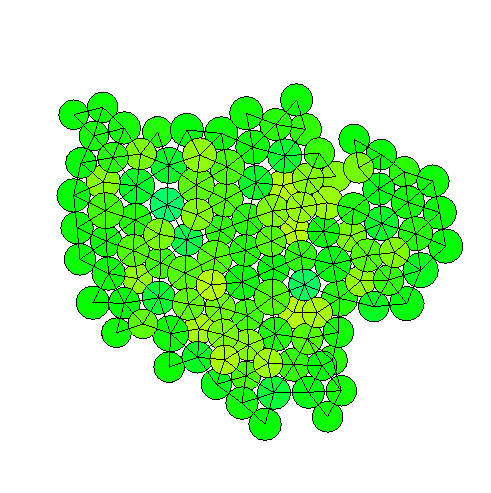

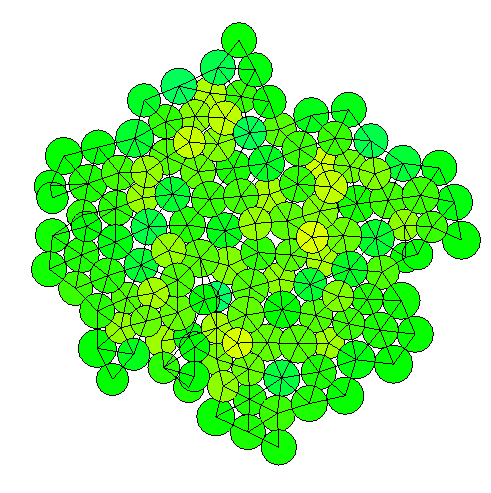


250


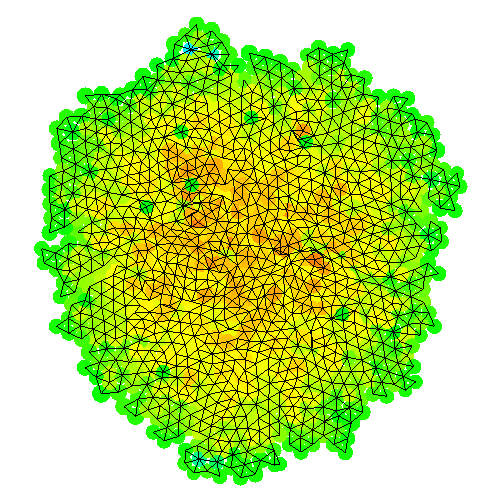

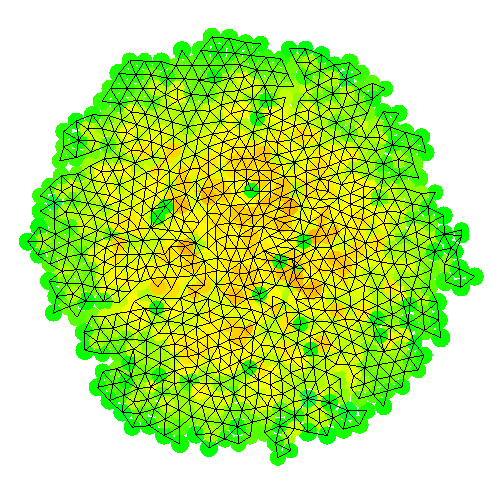

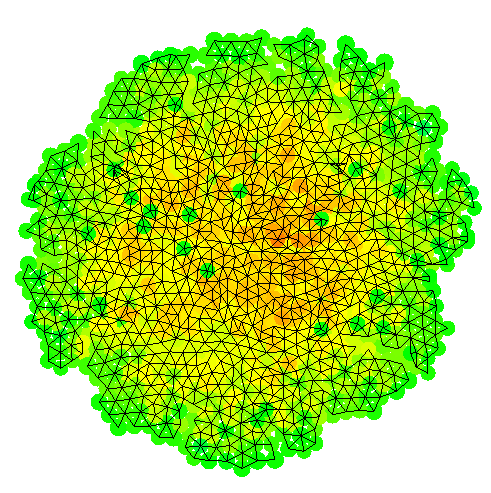

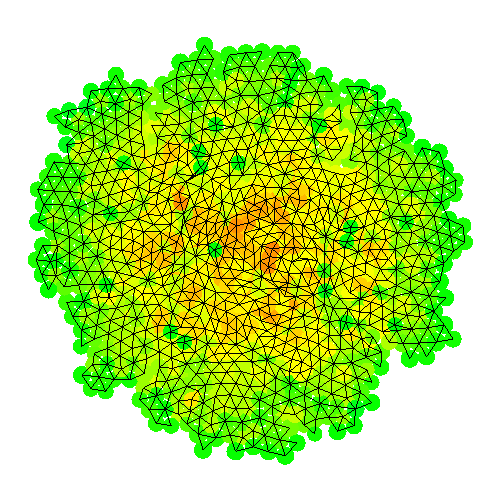

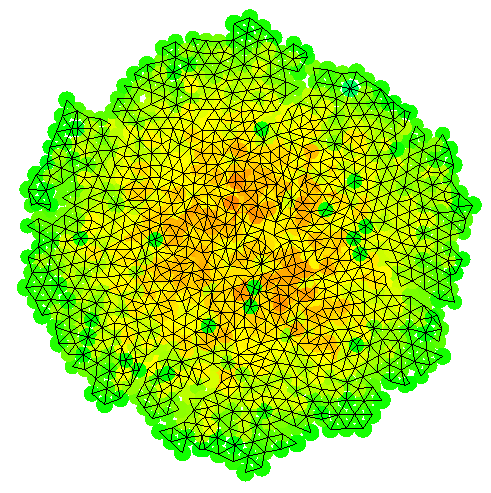

Supplement: Various samples [file rsos181127supp5.docx]
